# Lodox reveals the ideal body

**DOI:** 10.11604/pamj.2017.27.195.12822

**Published:** 2017-07-13

**Authors:** Lara Nicole Goldstein, Sameer Carim

**Affiliations:** 1 Division of Emergency Medicine, Faculty of Health Sciences, University of the Witwatersrand, Johannesburg, Gauteng, South Africa

**Keywords:** Ventilation, ideal body weight, critical care, emergency medicine

## Image in medicine

A 110 kg patient presented to the hospital emergency department after being involved in a motor vehicle collision. A full body LODOX^®^ (low-dose digital x-ray) was performed as part of the primary survey. The patient was subsequently intubated for airway protection. The utilisation of lower tidal volumes for ventilation of patients came to the fore with the ARDSNET publication. Despite the now well-known risks of ventilator-associated lung injury, patients with higher body mass indices who require ventilation in the emergency department are at increased risk of receiving inappropriately high tidal volumes. This Lodox image highlights that even though the patient's actual body weight was 110 kg and body mass index greater than 40, the lung size was equivalent to that of a 65 kg person. In addition, obesity can result in reduced lung volumes as well as reduced lung and chest wall compliance. This is exacerbated by the weight of the adipose leading to an increase in the work of breathing. It is a good reminder that in order to avoid deleterious, iatrogenic lung injury, we need to ventilate obese patients with tidal volumes scaled to their ideal body weight and mitigate the decreased chest wall compliance by considering starting with higher PEEP levels.

**Figure 1:**
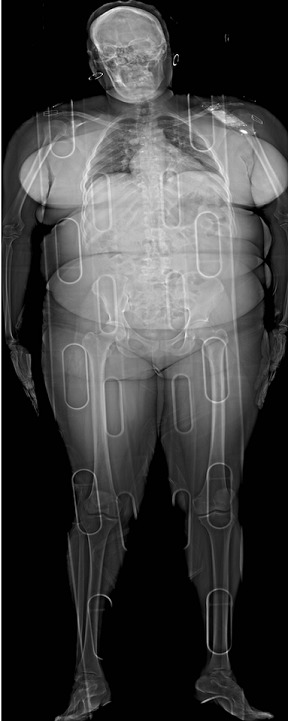
Full body lodox image

